# Zinc and Cancer: Implications for LIV-1 in Breast Cancer

**DOI:** 10.3390/nu4070648

**Published:** 2012-07-04

**Authors:** Bruce J. Grattan, Hedley C. Freake

**Affiliations:** 1 Department of Family Medicine, Stony Brook University Hospital Medical Center, Stony Brook, New York, NY 11597, USA; 2 Department of Nutritional Sciences, University of Connecticut, Storrs, CT 06268, USA

**Keywords:** zinc, zinc transporters, cancer, LIV-1, ZIP6

## Abstract

Zinc is a trace mineral which is vital for the functioning of numerous cellular processes, is critical for growth, and may play an important role in cancer etiology and outcome. The intracellular levels of this mineral are regulated through the coordinated expression of zinc transporters, which modulate both zinc influx as well as efflux. LIV-1 (ZIP6) was first described in 1988 as an estrogen regulated gene with later work suggesting a role for this transporter in cancer growth and metastasis. Despite evidence of its potential utility as a target gene for cancer prognosis and treatment, LIV-1 has received relatively little attention, with only three prior reviews being published on this topic. Herein, the physiological effects of zinc are reviewed in light of this mineral’s role in cancer growth with specific attention being given to LIV-1 and the potential importance of this transporter to breast cancer etiology.

## 1. Introduction

Nutrition plays a profound and increasingly recognized role in mitigating risk factors for chronic disease, with specific nutrients and the cellular responses they control emerging as mediators of key physiological events. Despite this fact, trace mineral research with regard to pathophysiology remains in its infancy. Zinc is a trace mineral which is vital for the functioning of numerous cellular processes, is critical for growth, and may play an important role in cancer etiology and outcome. Herein, the physiological effects of zinc are reviewed in light of this mineral’s role in cancer growth with specific attention being given to LIV-1 (ZIP6) and the potential importance of this zinc transporter to breast cancer etiology. 

## 2. Functions of Zinc

The trace mineral zinc is a requirement for all life on earth [1]. Despite being the 27th most abundant element, the physiological importance of zinc is unparalleled [1]. While less than for other minerals, the natural abundance of zinc is vast and an important determinant impacting its use in biological systems. Evolutionarily, the presence of adequate environmental exposure to zinc predicated its use in an array of biological systems. When considered against the backdrop of other micronutrients, zinc is unique in its functional diversity. 

There are four general biological roles of zinc, which include its structural, signaling, catalytic, and regulatory functions [2]. While the charge of this divalent cation determines both its reactivity and stability, its size determines where it can fit in biological systems. With regards to its catalytic functions, this mineral is intimately involved in the functioning of over 300 enzymes, the first of which (carbonic anhydrase) was so characterized seventy years ago [3]. Importantly, zinc is the only metal to be found in all six IUPAC classes of enzymes [1]. 

The coordination chemistry of this mineral enables its binding to an array of biological proteins and structurally, zinc is required for perhaps thousands of proteins. Intracellular concentrations of zinc exceed that of any other trace element and, in this milieu, zinc is critical for the functioning of greater than 3000 transcription factors [4]. It is the unique coordination chemistry and Lewis acidity of zinc that make it suitable for such widespread utilities [1]. In addition, its single valency and lack of different oxidation states favors stable interactions with proteins. Zinc, as a divalent cation, strongly interacts with electronegative atoms such as sulfur, oxygen and nitrogen, a property of its coordination chemistry that aids in protein-protein interactions [5]. 

The role of zinc in cell growth and division as well as basal homeostasis is of key importance. Zinc is required for the stabilization of the nucleic acids deoxyribonucleic acid (DNA) and ribonucleic acid (RNA) [6,7]. In fact, all RNA polymerases (I, II, III) are zinc metalloenzymes. Supplementation of zinc has been shown to promote DNA synthesis while depletion of this mineral inhibits DNA synthesis [8] and its presence in nucleoli has been known for over 50 years [9]. 

The unique DNA binding domains referred to as “Zinc Fingers” constitute a widely recognized example of the requirement of zinc for transcriptional processes within cells. The C_2_H_2_ zinc finger, consisting of two conserved cysteine and two histidine residues complexed with zinc is the DNA binding motif most commonly encoded by the human genome [10]. The DNA binding domains of nuclear receptors for such ligands as steroids, thyroid hormone, retinoic acid and vitamin D all possess similar characteristic complexes with four cysteine residues for each zinc cations [11]. Overall, zinc-binding proteins may constitute upwards of 10% of the human genome [12].

## 3. Zinc Transporters

The involvement of zinc in an array of physiological processes necessitates tight control of cellular zinc levels [13]. Not only is zinc a requirement for all cells, excessive concentrations of the mineral are known to be cytotoxic [5,14]. Zinc cannot passively diffuse through the cell membrane and requires transporters for its passage [15,16] and therefore control of cellular zinc homeostasis falls largely to the role of these zinc transporters and the regulation to which they are subject. Such transporters are regulated by both transcriptional and post-translational modifications [15]. There are two known families of zinc transporters, the Zrt-Irt-like proteins (ZIP family) and the Cation Diffusion Facilitator (CDF) family. The latter of which is often referred to as the ZnT family. Transporters of the ZIP family are responsible for uptake of zinc from outside the cell into the cytoplasm, while also playing a role in zinc efflux from subcellular organelles into the cytoplasm. Members of the ZnT family of transporters however possess an opposite role, functioning in the efflux of zinc from the cytoplasm out of the cell, as well as in the translocation of zinc from the cytoplasm into organelles, effectively diminishing cytosolic zinc concentrations [15]. 

Transporters within the ZIP family can be further demarcated as belonging to one of four subfamilies; ZIP-I, ZIP-II, gufA, or LIV-1 [15]. While “LIV-1” comprises an entire subfamily of zinc transporters, the literature often uses ZIP6 and LIV-1 interchangeably in reference to the specific transporter from which the subfamily’s name is derived. This transporter will be referred to as LIV-1 in this review. All ZIP transporters possess five to eight transmembrane spanning domains, with regions of concentrated cysteine and histidine residues, which may permit zinc specific binding [15]. While the precise mechanism is unknown, partially the result of a lack of crystallography data with regard to ZIP transporter structure, it is believed that the cationic nature of zinc enables its interaction with the electronegative cysteine and histidine residues directing the mineral towards its transporter. The essentiality of the histidine residues in the intracellular loop region (transmembrane domains IV and V) for zinc transport has been suggested based upon the results of site directed mutagenesis experiments [17]. Unlike most other minerals whose concentrations are homeostatically maintained by a single or a few transporters, zinc has a number of transporters. The multiplicity of zinc transporters suggests that distinctive features are retained for each. Each zinc transporter may be positioned so as to target this mineral to unique substrates and pathways requiring it. As proposed by Kagara *et al.* [18], there may be a specific chaperone molecule that transfers zinc imported through each zinc transporter to its functional target molecule. Furthermore, zinc itself may be an intracellular signal transducer [19].

## 4. Zinc Deficiency

Dietary intake patterns for zinc are still suboptimal, even in developed countries [20]. In the US currently 50% of men and women age 50 and older are consuming less than the RDA, 11 and 8 mg respectively, with 10% consuming less than one half of the recommended amount [21]. Likewise, a mere 18% of pregnant women worldwide have a zinc intake that meets the U.S. RDA [22]. Worldwide an estimated 2 billion may be zinc deficient [23]. Although the symptoms of overt zinc deficiency are seldom observed in developed nations, it is currently unclear whether this far-reaching pattern of suboptimal dietary zinc intake imparts increased risk for chronic disease, the leading cause of death and debilitation in developed countries. Overall, at least 20% of all humans are zinc deficient [24]. 

Owing to the widespread importance of this trace element, it can be expected that aberrations in zinc concentration have a profound effect on health, beginning at the cellular level. Indeed, research suggests that cancer may be one of the maladies of disrupted zinc status. For instance, zinc intake is inversely associated with colon cancer risk [25]. Although, currently, specific recommendations beyond the RDA cannot be made for cancer patients or those at high risk of cancer, modulating dietary intake or cellular utilization of this mineral may be a potential mode of intervention for preventing or treating this disease. There is evidence demonstrating that zinc deficiency can lead to DNA damage and the initiation of cancer. 

## 5. The Role of Zinc in Cancer

While many factors are involved in the etiology of cancer, it has been clearly established that diet significantly impacts risk of this disease [26,27,28]. The age-adjusted incidence of cancer in the US is 3 times higher than that in Asian countries, with immigrants to the US having increased risk for this condition [29,30]. This suggests critical roles for dietary and lifestyle factors.

The ubiquity of zinc in biological processes lends itself to the idea that aberrations in zinc status may play a significant role in cellular dysfunction, including the development and/or progression of cancer. Evidence in support of this idea is extensive. Zinc is known to be essential for cell proliferation [31,32], and is important for tumor growth [33,34,35]. The cell proliferating effects of growth factors are accompanied by an increase in the concentrations of labile zinc [32] and in the absence of zinc, cells are arrested in the S-phase, with cell proliferation being attenuated [31]. 

Work by Dutta *et al.* [36] has shown that addition of the chelator DTPA to the media reduced ^65^Zn uptake in GH3 rat pituitary tumor cells and reduced efflux when GH3 cells were pretreated with DTPA. Similar treatment using the primary counterparts to GH3 cells resulted in reduced uptake from the media, yet to the contrary, enhanced efflux when cells were pretreated with ^65^Zn. These findings were replicated using H4IIE cells and primary hepatocytes. Taken together these results suggest that cancer cells develop mechanisms to shut down zinc efflux and maintain intracellular concentrations when availability is reduced. This demonstrates the importance of intracellular zinc for rapidly dividing cancer cells. Experiments using shRNA against Znt-1, the only currently recognized plasma membrane zinc efflux transporter, have more recently demonstrated the requirement of Znt-1 for these effects [37].

## 6. Tissue Specificity of Zinc’s Association with Cancer

It has been shown that, * in vivo*, the growth of mammary carcinomas is suppressed following zinc depletion both in humans [38] and in mice [39,40]. However, these alterations in total zinc content are tissue specific changes rather than an observation associated with a generalized transformed state since malignancies of such tissues as the liver, [41] gallbladder, [42] prostate [43,44], lung, [45] cervix, as well as uterine myeloma [46], all are associated with a decrease in tissue zinc concentrations compared to their non-cancerous counterparts. Thus, although the overall levels of cellular/tissue zinc are altered in cancer, the modulation of specific zinc transporter expression is often observed. 

With regard to breast and prostate cancer, several comparative studies have provided evidence that altered zinc homeostasis occurs. For example, it is well established that zinc is vital for normal prostate function. In healthy individuals zinc concentrations in prostate are nearly 10-fold greater than in any other soft tissue [43,47]. However, the concentrations of zinc found in malignant prostate tissue are on the order of 75% lower than normal prostate [47,48]. Additionally, alterations in both cellular and serum zinc content have been observed in studies of breast cancer patients [49,50], where there is a 72% increase in tissue zinc concentration in comparison with normal tissues. This evidence regarding breast cancer is coupled with an observed reduction in serum zinc levels [51]. Likewise, not only do normal and malignant tissue types differ in zinc concentration, but also within individual patients, cancerous breast cells *tend* to accumulate more zinc than juxtaposed non-cancerous breast cells [52]. Breast cancer appears to be unique in its acquisition of zinc, suggesting the potential for this mineral’s involvement, and perhaps requirement, for breast malignancy. 

## 7. Association of Zinc Transporters with Cancer

As changes in cellular zinc concentrations are likely the result of alterations in the expression of zinc transporters, recent studies have been aimed at examining these differences. In prostate cancer, a potential mechanism for the dramatically diminished concentrations of zinc in malignant tissue is found with the downregulation of ZIP1 [47]. ZIP1 is known to be downregulated in prostate cancer [47] and zinc suppresses growth and induces cell death of prostate cancer cells via release of mitochondrial cytochrome c [53]. Conversely, it has been reported that zinc accumulation experimentally induced through over-expression of ZIP4, leads to enhanced progression of established pancreatic cancer [54]. ZIP4 expression in pancreatic tumors is elevated nearly 5.5 times the levels seen in the surrounding non-cancerous tissue [54]. Likewise, LIV-1, has been observed to be of importance for breast cancer [55,56], pancreatic cancer [57], cervical cancer [58], and prostate cancer [59]. Furthermore, both LIV-1 and ZIP10 are associated with breast cancer metastasis to lymph nodes, and may play a causal role [18]. 

Despite these interesting findings, a clear determination of whether these changes are a cause or an effect of the disease is unclear. While studies do vary, there appears to be a trend toward ZIP transporter upregulation in malignancies [57,59]. It has been hypothesized that the increased rate of proliferation of cancer cells may necessitate augmentation of cellular zinc uptake. The previously discussed evidence supports this hypothesis, as do the results of studies of zinc transporter silencing. The silencing of ZIP10 in breast cancer has been shown to diminish cellular capacity for migration, a key step in metastasis [18]. Comparable results have been demonstrated with LIV-1 in HeLa cells [58].

The induction of zinc transporter genes occurs through a variety of factors, many of which are recognized as contributing to tumorigenesis. Zinc transporters are subject to regulation by a variety of means including zinc itself, as has been demonstrated for Znt-1 [60,61] and ZnT-2 [61], as well as hormones [55], growth factors [62] and potentially cellular redox state [19,63]. Despite this knowledge, it is unclear as to whether zinc itself or zinc transporters are the actual mediators of malignancy-associated events [18]. The potential for zinc transporters to be direct mediators stems from the divergent roles these proteins may play. For instance, it has been suggested that LIV-1 may be vital for the functioning of matrix metalloproteinases or may even function directly as one itself [56]. As recently demonstrated by Lue *et al.* [59], the overexpression of LIV-1 was found to result in increased activity of MMP-2 and MMP-9. Such an increase in the enzymatic activity of MMPs was observed to result in cleavage of heparin binding epidermal growth factor, resulting in the constitutive activation of the epidermal growth factor receptor. Likewise, it has been proposed that zinc transporters such as LIV-1 may be mediators of key intracellular growth regulating signaling pathways such as the mitogen activated protein kinase pathway (MAPK) [58,64], which is often functioning aberrantly in transformed cells. 

## 8. The Association of Cellular Zinc Status with Cancer Development

While changes in the expression of zinc transporters have been observed in cancer models, zinc itself may serve a protective role against cancer in particular at the stage of initiation [65,66]. As excessive zinc is toxic, the sequestration of this mineral plays an important role in maintaining cell viability. Zinc, in addition to other heavy metals, is known to induce the expression of the cellular apo-protein thionein forming the metal-bound metallothionein (MT). The cellular stress sensor thionein is induced when over-accumulation of metals leads to zinc displacement from MT or when an accumulation of heavy metals exceeds the binding capacity of MT. This free zinc is then able to bind the metal responsive transcription factor (MTF-1), translocating it to the nucleus [67]. Once there, it binds to metal response elements (MRE) in the MT promoter, and induces expression to compensate for the heavy metal toxicity. This schematic has been supported by the discovery of the MRE consensus sequence TGCRCNC, to which MTF-1 binds [68]. Additionally, MTF-1 may be activated through phosphorylation induced by metal induced activation of pathways important to cell growth such as PKC, PI3K, or c-Jun *N* terminal Kinase (JNK) [69]. Moreover, MTF-1 has been shown to be critical for the DNA synthesis induced by epidermal growth factor (EGF) [70], which has a profound role in breast cancer [71,72].

Heuchel *et al.* (1994) demonstrated that the induction of metallothionein by zinc is due to direct binding of zinc to MTF-1 [73]. In turn, the MT protein has a variety of functions including the binding of heavy metals such as lead and cadmium, fulfilling a cellular detoxification role, while also sequestering zinc itself [74,75]. In relation to cancer, both MTF-1 and MT are over-expressed in radiation resistant tumors [76], perhaps supporting the importance of zinc acquisition for cancer growth. Despite the importance of MT for cell survival, concentrations in different cell lines differ by upwards of 400 fold [77]. MT may also serve as a cellular redox sensor and oxidants such as nitric oxide induce zinc release from MT [78]. Furthermore, zinc binding to MTF-1 leads to the induced expression of gamma glutamyl cysteine synthetase, the rate limiting step in glutathione production [79]. Likewise, the redox sensitivity of MT has been demonstrated to orchestrate zinc distribution, including zinc’s distribution to the critical antioxidant enzyme Cu/Zn Superoxide Dismutase (SOD). While this mechanism may at least partially mediate the antioxidant functions of zinc [80], MT itself has antioxidant functions and can scavenge superoxide anions, [81] quench hydroxyl radicals [82] and may be a surrogate for SOD in controlling superoxide levels [83]. In fact, antioxidants such as genistein, with known links to cancer prevention [84,85,86], may even exert their effect in part through induction of MT [87]. Overall, zinc and MT may be key regulators involved in the dysregulation observed in conditions of increased oxidative stress. 

Reduced concentrations of circulating antioxidants are associated with increased cancer risk [88]. It has been suggested that zinc deficiency may increase one’s risk for cancer in part through an elevation in the levels of free radicals and an overall state of increased oxidative stress [89]. As a Lewis acid, zinc possesses antioxidant functions in its capability of scavenging reactive oxidant species through acceptance of electrons [1]. Again unique to zinc is this mineral’s ability to function as a potent antioxidant while concurrently lacking the ability to foster free radical production as a pro-oxidant [1]. This is contrary to the known pro-oxidant effects of other antioxidant nutrients such as vitamin C [90] as well as the alternative (oxidizing) redox states of other transition metals such as copper and iron [91]. In particular, zinc is critical for cellular antioxidant defenses as part of Cu/Zn SOD, enabling the conversion of superoxide radicals to the non-radical hydrogen peroxide, which is also known to induce MT [92]. The structural integrity of Cu/Zn SOD is reliant upon zinc, although its catalytic function necessitates the use of copper. 

The role played by dysregulation of Nuclear Factor Kappa B (NFκB) in tumor development is now recognized [93]. In models of zinc deficiency, the functioning of Cu/Zn SOD in response to NFκB is impaired [94]. It has been suggested that the cellular response to oxidative stress may involve the oxidant-mediated release of protein-bound zinc and alterations in the labile zinc pool with subsequent apoptosis [95,96,97]. It has also been shown that the addition of zinc to breast cancer cells inhibits NFκB, which is constitutively activated in this disease and leads to a more aggressive, hormone independent phenotype [98,99]. In addition, downregulation of NFκB *in vivo* has been shown to increase cancer cell susceptibility to the apoptotic effects of TNF-α [100] while also minimizing the metastatic capability of these cells through modulation of growth factors and cytokines. In particular, the inhibition of vascular endothelial growth factor (VEGF), interleukin-8, interleukin-6, and matrix metalloproteinase-9 production is noted [101,102]. In prostate cancer, the addition of zinc has been associated with sensitization to cytotoxic agents [99].

Zinc has been implicated in mediating apoptotic cell death [103]. Both indirect and direct apoptotic effects of zinc have been demonstrated in cancerous cells. Evidence suggests that zinc induces cell growth arrest at G2/M in a dose dependant manner [48]. This indirect effect of zinc has been attributed to the role of this mineral in inducing the expression of p21, a cyclin-dependant kinase inhibitor known to govern cell progression at this phase [48]. Moreover, zinc may have a more direct effect on cell death. Studies treating prostate cancer cells with physiological concentrations of zinc have determined that this mineral fulfills its apoptotic functions through a mitochondrial effect [53] causing nearly immediate release of cytochrome c from the mitochondrial inner membrane. This released cytochrome c activates the caspase pathway responsible for the initiation of apoptosis. Experiments involving isolated mitochondria have found that zinc functions in a direct manner causing the release of cytochrome c in as little as ten minutes [53]. Even minute alterations in cellular zinc flux may stimulate apoptosis. Zinc status has been implicated in cancer risk with offspring malignancy being one such example. Paternal zinc deficiency in rats has been shown to be a contributing factor in pup cancer, leading to increased oxidative damage to testicular cell DNA [104]. This has been shown to be the result of both decreased activity of zinc dependent antioxidant systems such as SOD as well as increased iron load [105]. Furthermore, the offspring of zinc deficient Rhesus monkeys exhibit a greater incidence of chromosomal breaks [106]. Zinc is also involved in epigenetics, whereby heritable changes in gene expression occur without changes in DNA sequence [107,108]. At this level, zinc deficiency results in decreased DNA and histone methylation [109], which is attributed to the fact that histone lysine methyltransferases and histone deacetylases are zinc dependent enzymes [110,111,112,113]. Histone deacetylase inhibitors have been shown to reactivate the estrogen receptor (ER) in estrogen receptor negative (ER−) breast cancer cells [114]. 

## 9. Zinc and IGF-1

One likely partial explanation for the growth restriction seen in zinc deficiency is the suppression of insulin-like growth factor 1 (IGF-1) [115]. IGF-1 is diminished in a zinc deficient state and zinc supplementation has been demonstrated to increase levels of this growth factor [116]. Zinc is needed for IGF-1 production in addition to this factor’s binding to the IGF-1 receptor (IGF1-R). Similarly, it has been demonstrated that zinc activates the mammalian target of rapamycin (mTOR) in a manner independent of both amino acids and insulin [117]. Since mTOR is a regulator of protein synthesis, lack of activation by zinc is an additional pathway through which zinc deficiency may impede growth. The effects of zinc on the mTOR pathway overlap with this mineral’s effects on IGF-1 signaling. The effectiveness of mTOR inhibitors in cancer treatment [118,119] may be related to changes in cellular zinc levels.

Zinc status may influence IGF-1 signaling, which may in turn affect not only systemic growth, but cancer risk. The Health Professionals Follow-Up Study [120] found a correlation between elevated doses and/or long-term use of supplemental zinc and increased risk for cancer, specifically prostate cancer. However, Gonzalez *et al*. [121] found a relationship between high intake of supplemental zinc intake (>15 mg) and reduced risk of advanced prostate cancer, despite no reduction in absolute prostate cancer risk being observed between high *vs*. low dose. Similarly, others have found a negative correlation between zinc intake assessed near diagnosis with prostate cancer and risk of prostate-specific mortality [122]. Given the known influence of zinc on modulation of IGF-1 concentrations [116], supplemental dietary intake of zinc may have the effect of stimulating cancer initiation and/or growth. Elevated IGF-1 is related to an increased risk of developing several cancers including that of the breast [123], prostate [124], and colon [125]. IGFs have been shown to have mitogenic, transforming, and antiapoptopic properties, especially when coupled with other growth factors [126]. IGF-1 also promotes angiogenesis and thus may be a key target in the prevention of cancer cell extravasation and metastasis [127,128]. Likewise, IGF-1 has been demonstrated to induce the expression of the ER, while estrogens synergistically enhance IGF-1R mediated signaling [129]. Furthermore, numerous studies have linked alterations in IGF-1 with resistance to cytotoxic agents [130,131,132] and indeed inhibitors of these pathways have been developed for target cancer therapy [133].

In breast cancer, local IGF-1 production is mediated by surrounding stromal cells [134]. In addition to possessing the type I IGF-1R, through which the effects of IGF-1 are mediated, MCF-7 cells also produce and secrete IGF-1 binding proteins, which modulate the actions of IGF-1 and further supporting a role for local growth factors as a mediator of cancer cell growth in models used to study this disease [135]. This paracrine control of cell proliferation as mediated through the induction of IGF-1 by estradiol in MCF-7 cells has been known for nearly 30 years [136,137]. More recently, insulin and IGF-1 have been demonstrated to induce the expression of the zinc transporter LIV-1 in ER positive breast cancer cells [62] and the regulation of this zinc transporter may be important for invasiveness and estrogen-independent cell growth [64]. 

However, IGF-1 may be more important for hormonally independent breast cancers, since a blocking antibody to the IGF-1R in MCF-7 cells does not impede growth whereas this same antibody induces growth retardation in the ER- cell line MDA-MB-231 [138]. This has been demonstrated both *in vivo* [139] as well as *in vitro* [140]. Thus, while losing hormonal responsiveness, growth is maintained by growth factors rather than systemic hormones. 

While zinc deficiency may decrease cancer risk through reducing IGF-1, such a deficient state may increase the activity of aromatase (CYP19) [141], an enzyme required for the conversion of androgens, both testosterone and androstenedione [142], to estrogens. While aromatase is expressed in both normal and transformed breast cells, increased expression is a common finding in malignant cells, enabling the provision of estrogen needed for proliferation [143]. Not only does zinc deficiency increase the activity of aromatase, zinc deficiency may result in greater concentrations of the ER [141]. 

## 10. Zinc and Protooncogenes

### 10.1. P53

P53 is a well known apoptotic mediator [144] that also plays an important role in stimulating cell cycle arrest induced by DNA damage [144]. Mutations in the P53 have long been recognized to be prevalent in malignancies. In fact, it is estimated that P53 gene (TP53) mutations occur in nearly 50% of tumors [145]. Just as zinc is necessary for both RNA and DNA stability, [6,146] this mineral is known to be a critical structural component of P53 [147] and it has been shown that P53 binds DNA through a zinc coordinated domain. Indeed, it has been further demonstrated that the preponderance of P53 mutations occur specifically in such DNA binding regions. The maintenance of basal concentrations of P53 occurs through MDM-2 (Mouse Double Minute-2) mediated ubiquitination [148]. To balance this action, HAUSP/USP7, an ubiquitin hydrolase, in turn regulates MDM2 activity and therefore P53, while the deubiquitinating enzyme USP7 works to augment P53 levels through inhibition of its degradation [149]. Zinc directly impacts P53 binding, such that zinc deficiency blocks the formation of the functional P53-DNA complex necessary for transcription of downstream genes [4,150]. In fact, P53 protein content is actually augmented during zinc deficiency [65]. This increase may be of a dysfunctional P53 molecule, which may result from a negative feedback response mediated by diminished transcription of P53-regulated genes in order to sustain its activation. P53 concentrations may also be increased as a result of the DNA damage promoted by zinc deficiency. This is consistent with evidence demonstrating that there is an increase in the expression of apyrimidic endonuclease (APE/Ref-1) under conditions of cellular zinc deprivation. Thus, the need to repair nucleotide damage, the accumulation of which is a stimulus for P53 activation, may also be zinc mediated [4]. 

A relationship also exists between P53 and the ER. Over-expression of P53 prevents the recruitment of ER-α to activator protein-1 (AP-1) and represses transcription of breast cancer associated gene-1 (BRCA1) [151]. The *BRCA1* gene encodes a transcription factor involved in recombination and DNA repair and reduced levels of *BRCA1* as well as mutations in its sequence significantly increase susceptibility to breast and ovarian cancer [152]. Located at 17q21, *BRCA1* is a tumor suppressor gene that repairs double strand breaks through association with Rad51 protein [153]. Thus within this paradigm, zinc deficiency may increase estrogen through augmenting aromatase activity, while decreasing the tumor suppressing capability of P53, and increasing the expression of a nonfunctional P53, which in turn may repress the activity of BRCA1. 

### 10.2. KLF-6

Aside from P53 and BRCA1, zinc has also been demonstrated to be required for the action of another tumor suppressor protein, Kruppel-like factor 6 (KLF6), a zinc-containing protein that is also inactivated in a variety of cancers [154]. In the malignant state, it has been demonstrated that KLF6 binds cyclin D1 thereby preventing its binding to cyclin-dependent kinase 4 (cdk4), leading to growth arrest. The converse has also been demonstrated [154]. Experiments using short interfering ribonucleic acids (siRNA) to *KLF6* illustrate that loss of KLF6 results in restoration of cyclin D1/cdk4 complexes and the continuation through the cell cycle [154]. This adds to the pattern of deregulation observed in cancerous cells and an important role of zinc in these processes. While a decrease in cellular zinc is observed among prostate cancer cells, these cells likewise experience both mutations in *KFL-6* as well as loss of the expression of this transcription factor [155]. Such mutations may be secondary to KFL-6’s requirement for zinc. 

Overall, these examples give strong support for the idea that cellular zinc is not only tightly controlled, but that modulation of such control may be therapeutically beneficial in relation to cancer treatment. As has been previously described, this maintenance of cellular homeostasis is zinc transporter mediated, and changes to the amount and or activity of zinc transporters plays a significant role in cell viability since excess zinc as well as diminished concentrations of this mineral are both highly damaging and may lead to cell death. In cancerous cells, zinc transporters may be up-regulated as a means of maintaining cellular zinc homeostasis under conditions of increased requirement of this mineral, necessitated by the enhanced proliferation of transformed cells. Amid this elevated zinc flux, it can be presumed that other changes to malignant cells such as the modulation of zinc sequestration or transporters may enable them to avoid over-accumulation of zinc and its resulting cytotoxic effects. Thus, maintenance of cellular zinc homeostasis is not solely a requirement of normal healthy cells, but of transformed cells as well. Clearly zinc is a key player in malignancy and select transporters of zinc may emerge as important therapeutic targets in patients with established disease. In totality, the importance of this divalent cation in the etiology of cancer is becoming increasingly clear.

## 11. Zinc and Immune Functioning

Chronic inflammation is a fundamental aspect of cancer etiology [156,157,158] and so the immune system plays a pivotal role in cancer prevention and prognosis [159,160,161]. Aside from the general importance of zinc at the cellular level, the immune system is particularly reliant on adequate zinc nutrition [1]. There are several lines of evidence linking zinc status will immune function relevant to cancer. Nearly 20 years ago, Consolini *et al.* observed that patients with acute lymphocytic leukemia experienced reductions in plasma zinc concentrations at disease onset, with a return to normal zinc status upon remission [162]. Similarly, rapid reductions in plasma zinc concentrations are known to occur as part of the acute phase response [163] and this has been suggested to be the result of IL-6 mediated upregulation of ZIP14 [164]. Similarly, zinc is required for antigen-dependent T-cell activation [165]. *In vivo*, treatment with LPS reduced zinc concentrations in dendritic cells as well as LIV-1 levels, overall resulting in impaired dendritic cell maturation [166]. Zinc may also have anti-inflammatory effects suggested by the fact that zinc treatment attenuates monocyte release of proinflammatory cytokines (TNF-α) and IL1-β) [167]. Similarly, in T cells, zinc may be a critical signaling molecule stimulating PKC activation [168]. The observation of elevated glucocorticoids with concordant thymus atrophy in zinc deficient animal models [169,170] suggests that inadequate zinc levels may lead to systemic immunosupression. It is known that cancer incidence increases with aging [171] and aging is associated with both increased incidence of zinc deficiency as well as immunosenescence [172]. The relationship between zinc deficiency and impaired immune regulation of tumor growth may be causal given that Th1 cells have an established role in tumor suppression and animal models of zinc deficiency have been observed to experience reductions in Th1 axis cytokines including IL-2 and interferon γ [173]. 

## 12. LIV-1

The gene encoding the zinc transporter LIV-1 (SLC39A6), also known as *Ermelin* and *ZIP6*, is located on chromosome 18q12.2 (NCBI database). This gene was originally identified in the human breast cancer cell line ZR-75-1 as a 4.4 KB mRNA that was induced by estrogen treatment [55,174]. Later sequence analysis showed it to be part of the ZIP-family, predicting a role in cellular zinc homeostasis [56]. *In vitro* studies using ZR-75-1 have revealed a 4-fold induction of LIV-1 mRNA with estrogen treatment [174]. 

Work with another human breast cancer cell line, MCF-7, has demonstrated that LIV-1 expression is induced by estradiol, IGF-1 and IGF-2, as well as by insulin [175]. Additionally, in MCF-7 cells the combined administration of insulin and estradiol was shown to induce LIV-1 expression above the level observed with each hormone individually [62]. Likewise, simultaneous treatment of MCF-7 cells with physiological concentrations of IGFs and estradiol resulted in a similarly elevated expression of LIV-1 [126]. These results imply a synergistic role for these hormones (insulin + estradiol and IGF + estradiol) in the induction of the LIV-1 gene.

To a lesser degree, other steroid hormones such as 5α-dihydroxytestosterone, dexamethasone, and progesterone also induce expression of LIV-1 [55], although synergy has not been reported between insulin and these other hormones [55]. Given the known roles of estradiol, insulin, and IGFs in breast cancer etiology as well as the role of zinc in the regulation of aromatase and protooncogenes, LIV-1, may play a critical role in maintaining zinc homeostasis and therefore be vital to the development and progression of breast cancer. As such, it represents a potential point of focus for assessing breast cancer therapy, response to therapy, and risk for metastasis. However, to date such investigations have been limited to cell culture studies. 

### 12.1. LIV-1 and Breast Cancer

The expression of LIV-1 has been suggested to be of importance to breast cancer etiology [55,56]. However, in relation to cancer, the role of LIV-1 is unclear. Some studies correlate LIV-1 expression with a more aggressive cancer phenotype and increased likelihood for metastasis to lymph nodes [176,177]. In contrast, other evidence suggests this transporter is associated with a more favorable prognosis [18,178,179]. 

LIV-1 has been closely associated with the estrogen receptor (ER) [176] and there are a variety of structural features unique to LIV-1 that are not observed in other zinc transporters. To date the only mammalian zinc transporters in which a metalloproteinase motif has been identified are LIV-1 [56] and ZIP4 [180]. Metalloproteinases are a group of zinc requiring proteolytic enzymes, which are directly involved in the metastasis of cancer cells to lymph nodes [181]. In addition to possessing the necessary structural motifs, Taylor *et al.* [56] demonstrated that LIV-1 localizes to the plasma membrane lamellipodiae in a fashion similar to matrix metalloproteinases. Thus, LIV-1 may play a dual role, functioning in both cell growth via its operation as a zinc transporter, as well as metastasis of breast cancer through its association with matrix metalloproteinases [59]. Studies have shown that expression of LIV-1 is statistically associated with lymph node involvement of ER positive metastatic breast cancer. In small tumors, 92% of lymph node positive patients expressed LIV-1, whereas 77% of lymph node negative patients failed to express LIV-1 [177]. Despite these findings, a causal relationship between LIV-1 and metastasis to lymph nodes has not been fully established, although structurally the potential for such a relationship is noticed. 

Despite the association with matrix metalloproteinases [56,59] some studies have demonstrated that LIV-1 expression in breast cancer patients is correlated with a significantly better outcome [178]. Work by McClelland *et al.* (1998) demonstrated that detection of LIV-1 can be used to signify a patient’s positive response to endocrine therapy [182]. Due to its induction by estradiol, *LIV-1* has been proposed to be a marker gene to assay ER+ breast cancer *in vivo*. Several studies, beginning in 1995 [183], have demonstrated a correlation between ER positive status, LIV-1 and nodal involvement [177,182]. The progesterone receptor is a classic estrogen responsive gene traditionally used to assess ER status, however the assessment of LIV-1, both alone or in combination with positive ER status, has been shown to represent an improved prognostic marker of relapse free survival [56,178]. 

While *LIV-1* is a marker gene for such conditions, ER positive breast cancer itself represents a more favorable prognosis than the ER negative phenotype. This is simply from the standpoint that the presence of the ER maintains cell responsiveness to endocrine therapy such as with the selective estrogen receptor modulator (SERM), Tamoxifen, and, more recently, aromatase inhibitors [184]. ER negative breast cancer is not susceptible to this treatment [185]. Interestingly, the sensitivity of LIV-1 expression to estradiol is supported by the fact that treatment of MCF-7 cells with trans-hydroxytamoxifen, which exerts both agonistic and antagonistic actions, increases LIV-1 expression [175]. LIV-1 is not the only estrogen regulated zinc transporter, since the expression of ZnT-3 is inhibited by estrogen [186]. As members of the ZnT family function in zinc efflux, this is consistent with a role for estrogen increasing cellular zinc concentrations. 

The use of LIV-1 as a prognostic marker may extend beyond the simple demonstration of the existence of the ER. In fact, McClelland *et al.* [187] have demonstrated that treatment using the pure antiestrogen ICI 182780 (Fulvestrant), which has the result of decreasing estrogen receptor mRNA concentrations, had no measurable effect on LIV-1 mRNA expression despite decreasing estrogen receptor protein content, implying multiple regulatory pathways for this zinc transporter. Additionally, this may underscore the importance of this zinc transporter to breast cancer cell viability [187].

Aside from its association with MMPs, LIV-1 expression is also associated with other pathways of growth and metastasis, such as MAPK. LIV-1 is not only overexpressed in the human cervical cell line HeLa compared with normal cervical tissue, but suppression of LIV-1 by means of siRNA diminishes the invasive capability of these cells through a mechanism targeting the ERK1/2-Snail/Slug pathway [58]. In this regard LIV-1 suppression is accompanied by downregulation of both p44/42 MAPK and its phosphorylated form [58]. These data suggest a detrimental effect of LIV-1 expression contrary to its association with ER status. 

While McClelland’s work utilized biopsied samples [182], in MCF-7 cells, LIV-1 has been shown to be downregulated by Fulvestrant [176]. Treatment of T47D cells with Fulvestrant decreased zinc accumulation but did not alter LIV-1 abundance, despite the importance of LIV-1 in zinc accumulation being directly demonstrated [188]. This illustrates that while LIV-1 may be estrogen regulated, the estrogen signals responsible for its expression may not be following a traditional steroid hormone response element pathway. To that end, despite the causal role of estrogen in the progression of breast cancer, and approximately 80% of breast cancer cases in Western countries being (ER+) [189], a substantial number of breast cancers cases do not exhibit positive ER status [190]. Moreover, approximately 33% of genes which are in fact ER regulated, fail to contain ERE sequences, even those highly associated with breast cancer risk such as *BRCA1* [191].

The classic scenario for estrogen signaling, due to its steroidal nature, is the capability of this hormone to pass through the plasma membrane where it binds to its cytosolically located ER. Upon binding, the ER-ligand complex translocates into the nucleus, dimerizes and binds to the estrogen response element (ERE), a unique sequence on DNA [192]. However, the ER may also interact with other molecules such as activator protein-1 (AP-1) or the zinc finger transcription factor SP-1 [193]. For genes containing GC-rich promoter sequences it has been shown that ER initiates their transcription through interacting with Sp1 [193,194,195] rather than directly binding to the promoter itself [196]. 

The receptor to which estradiol binds, is also an important determinant of this hormone’s cellular effects. The structure of ER-α is such that it possesses a hormone binding domain, a DNA binding domain and two transcriptional activation domains (AF-1 and AF-2) [138]. Ligand binding to ER-α alters the conformation promoting either the release of co-repressors and/or the binding of co-activators leading to the transcription of genes. Site directed mutagenesis studies have demonstrated that ERs lacking the AF-2 domain are not activated by their intrinsic ligand, but rather are activated by IGF-1 and EGF. Thus, AF-2 is required for the ligand-dependent activation of the ER [138]. Recently, other signaling modalities of estrogen have been discovered, wherein the ER may maintain an association with the plasma membrane and not function directly as a transcription factor. By doing so, the ligand activated ER is able to interact with receptor tyrosine kinases including EGFR and IGF-1 or Src (a non-receptor tryrosine kinase) and exerts its effects through phosphorylation cascades rather than direct transcriptional activity [192,193]. Interestingly, *LIV-1* has been demonstrated to be a downstream target of Signal Transducer and Activator of Transcription-3 (STAT3) [197], a member of the Janus Kinase pathway (JAK). As *LIV-1* may be induced by EGF and TGF-α in addition to its induction by estradiol, it is therefore possible that *LIV-1* is not induced by estradiol via traditional mechanisms for this steroid hormone involving its binding to ERE, but rather through phosphorylation by the kinases activated by these hormones and growth factors. Evidence for this stance includes the fact that there is a synergistic induction of *LIV-1* by both estradiol and IGF-1. Thus, crosstalk between membrane associated ligand-bound ER may interact with IGF-1 initiated pathways to stimulate a variety of phosphorylation events [138], all of which may converge on STAT3. *LIV-1* is a downstream target of STAT3, which is activated by means of a phosphorylation cascade initiated following EGFR stimulation. Moreover, no study to date has definitively elucidated an ERE for *LIV-1*, suggesting the potential for non-genomic estrogenic signaling as a means of regulating *LIV-1* expression. In breast cancer approximately 10% of ER-α negative patients respond to endocrine therapy while nearly 30% of ER-α positive patients do not [198]. Therefore, newer treatments which do not solely rely upon the ER are of great importance. 

### 12.2. LIV-1 and Metastasis

LIV-1 has been implicated in metastasis beyond its association with MMPs [59]. The epithelial to mesenchymal transition (EMT) is a consistently observed phenomenon that is a vital aspect of embryogenesis as well as cancer progression [64]. During the EMT, cancer cells lose their adhesion and begin the process of metastasis. A key observation in studies investigating the EMT in malignancy has been that there is a loss of the cell adhesion protein E-Cadherin. E-Cadherin is a calcium dependent transmembrane glycoprotein and its reduced expression has been associated with breast cancer metastasis [199,200]. STAT3 and the zinc finger transcription factor Snail regulate E-Cadherin expression through transcriptional repression [64]. One study, for instance, demonstrated that 67 percent of Snail positive tumors displayed reduced E-Cadherin and 100% tested positive for metastasis [201]. It has been demonstrated that, in response to *LIV-1* silencing, E-Cadherin is down-regulated [179]. In zebrafish embryos, LIV-1 is dependent on Snail for its activity. Evidence for this claim is such that co-injection of LIV-1 mRNA into embryos deficient in Snail was not capable of abrogating the defects observed during Snail deficiency. Likewise, the injection of a morpholino against Snail globally eliminated the effects of LIV-1 on EMT [197]. In breast cancer, LIV-1 and E-Cadherin are positively correlated, such that LIV-1 is negatively associated with EMT [202]. To the contrary, in pancreatic cells, LIV-1 expression has been observed to be associated with both tumor size and lymphatic infiltration [57]. Contrary to the findings that LIV-1 plays an inhibitory role with regard to the EMT in T47D cells, the expression of this transporter has been shown to promote the EMT in prostate cells [59] and is associated with cervical cancer invasiveness [58] as well. Clearly these findings demonstrate divergent, tissue specific, regulatory pathways for this zinc transporter. 

MTA3 (metastasis associated factor 3) is a member of the Mi-2/NuRD transcriptional corepressor, which is a downstream target of ER-α [203] and it has been shown that MTA3 downregulates Snail [204]. Despite this notion, studies have failed to demonstrate a correlation between Snail mRNA and ER-α protein expression [204]. Similar to conditions of zinc deficiency, Snail has been demonstrated to downregulate the promoter mainly responsible for aromatase transcription [205]. Thus, a situation is presented whereby activation of the ER by its cognate ligand may act through MTA3 to decrease *in situ* estrogen production [204]. This may affect zinc transport given that in response to estradiol, LIV-1 is upregulated [206] and Znt-3 is inhibited [186] at least in brain. In breast cancer, estrogen signaling and zinc transport is intimately linked via multiple pathways [188]. 

It may be that not only does augmentation of LIV-1 in some cancer cells allow for the increased zinc flux needed to maintain their rapid proliferation, but through an increase in Snail, such overexpression of LIV-1 may decrease E-Cadherin and promote the cell’s metastatic potential. This is in contrast to breast cancer cells in which LIV-1 expression is positively correlated with E-Cadherin expression [179]. Necessitated by the fact that a majority of cancer deaths are attributed to metastatic disease [207], there is great interest in understanding the regulation of cellular adhesion molecules.

## 13. Conclusions

Clearly, proper cellular zinc status profoundly affects cellular health and has numerous roles in cancer initiation, progression, termination, and potentially prevention. The interaction between zinc status, zinc transporter expression, and immune function is interwoven with cellular signaling pathways providing multiple mechanisms for the role of zinc in cancer. There are direct effects of zinc on tumor cells, changes in the expression of zinc transporters in cancer, a role for zinc in the stability and functioning of intracellular machinery related to cell division, and effects on immune function, tumor surveillance and apoptosis. Zinc transporters play important roles in cancer etiology with LIV-1 being the newest addition to this growing network of effects. LIV-1 may be important for both cancer prognosis and treatment, especially for breast cancer, and should be more thoroughly investigated. Research into LIV-1 may yield important information regarding signaling between the ER and the EGFR, as well as the changes that occur in making breast cancer cells both drug resistant and capable of metastasis.
